# Supplementary figures and images for: Discrete mechanical model of lamellipodial actin network implements molecular clutch mechanism and generates arcs and microspikes
Source: PLoS Comput Biol. 2021 Oct 18;17(10):e1009506. doi: 10.1371/journal.pcbi.1009506 (PMC8553091; doi:10.1371/journal.pcbi.1009506)

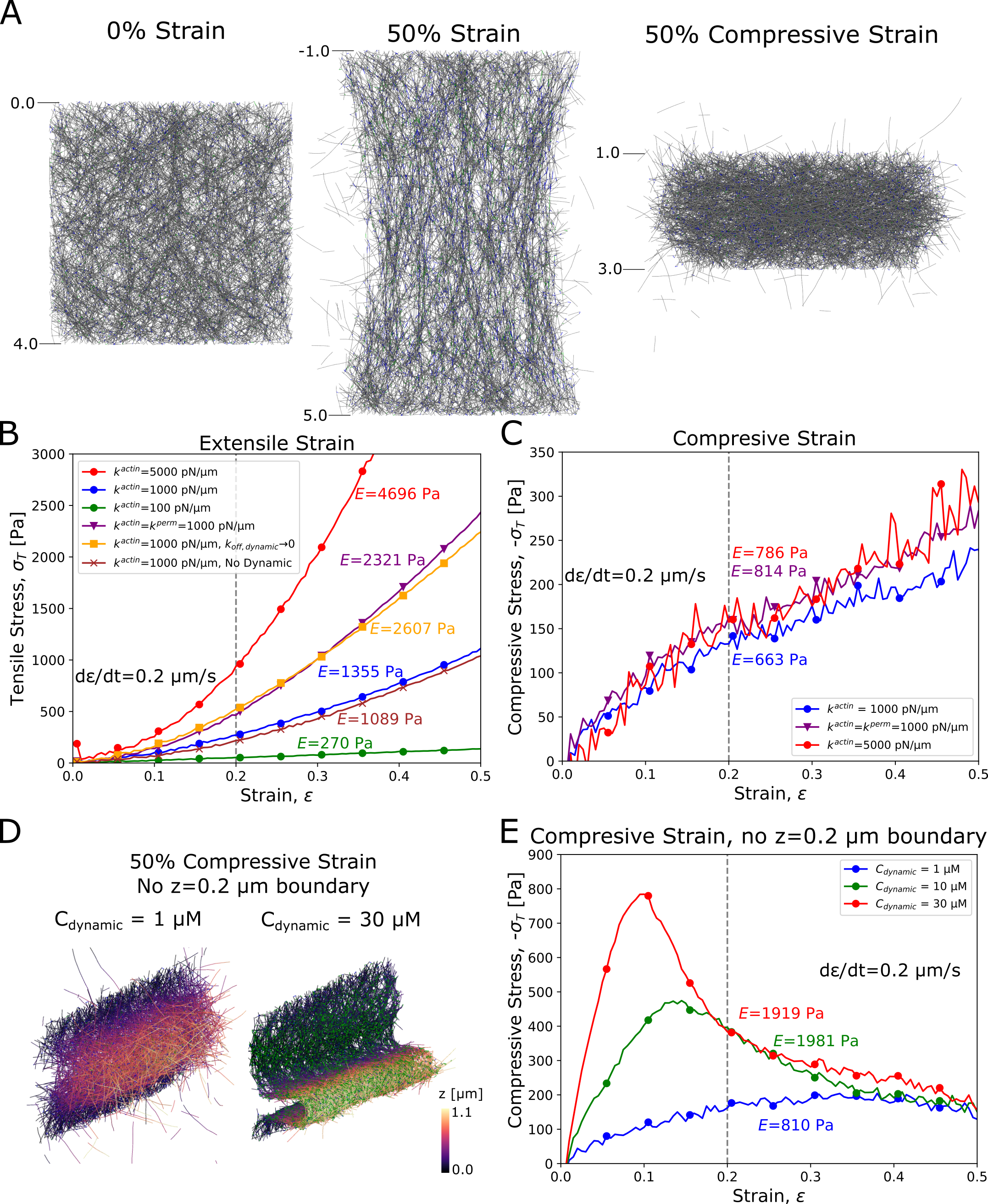

Supplement: S1 Fig — (A) A sample of the actin network taken close to the leading edge from a simulation at κFA = 1.0 and uniform pulling is put under either extensile or compressive strain up to a value of 50% at a strain rate of 0.2 μm/s. (The diffusing filaments escaping the network are filaments that got disconnected when the network was cut.) Blue line segments represent permanent crosslinkers while green line segments represent dynamic crosslinkers. (B) Tensile stress σT versus strain measured by extension of the network. The elastic modulus is calculated by dividing σT at a strain of 0.2 by the strain. We investigate the effect of varying the actin bond spring constant kactin, permanent crosslinker spring constant kperm, and the dynamic crosslinker dissociation rate kdynamicoff on the elastic modulus. Other parameters used as in Table 1, with the blue curve having all parameters (including kactin) as in Table 1. Slight prestress in the kactin = 5000 pN/μm curve can be seen as a non-zero value of σT at zero strain. (C) Tensile stress σT versus strain measured by compression of the network. Boundary confining forces at z = 0 and z = 0.2 μm prevent the thin sheet from buckling along the z direction. Elastic modulus values measured for compression are lower than the same conditions as in (A) and less dependent on the values of kactin, kperm. (D) Networks under compression without the stabilizing effect of a repulsive boundary at z = 0.2 μm exhibit buckling. Increasing Cdynamic results in more cohesive networks. Color indicates z value. (E) Tensile stress σT versus strain measured by compression of the network without a repulsive boundary at z = 0.2 μm. (TIF) [file pcbi.1009506.s001.tif]

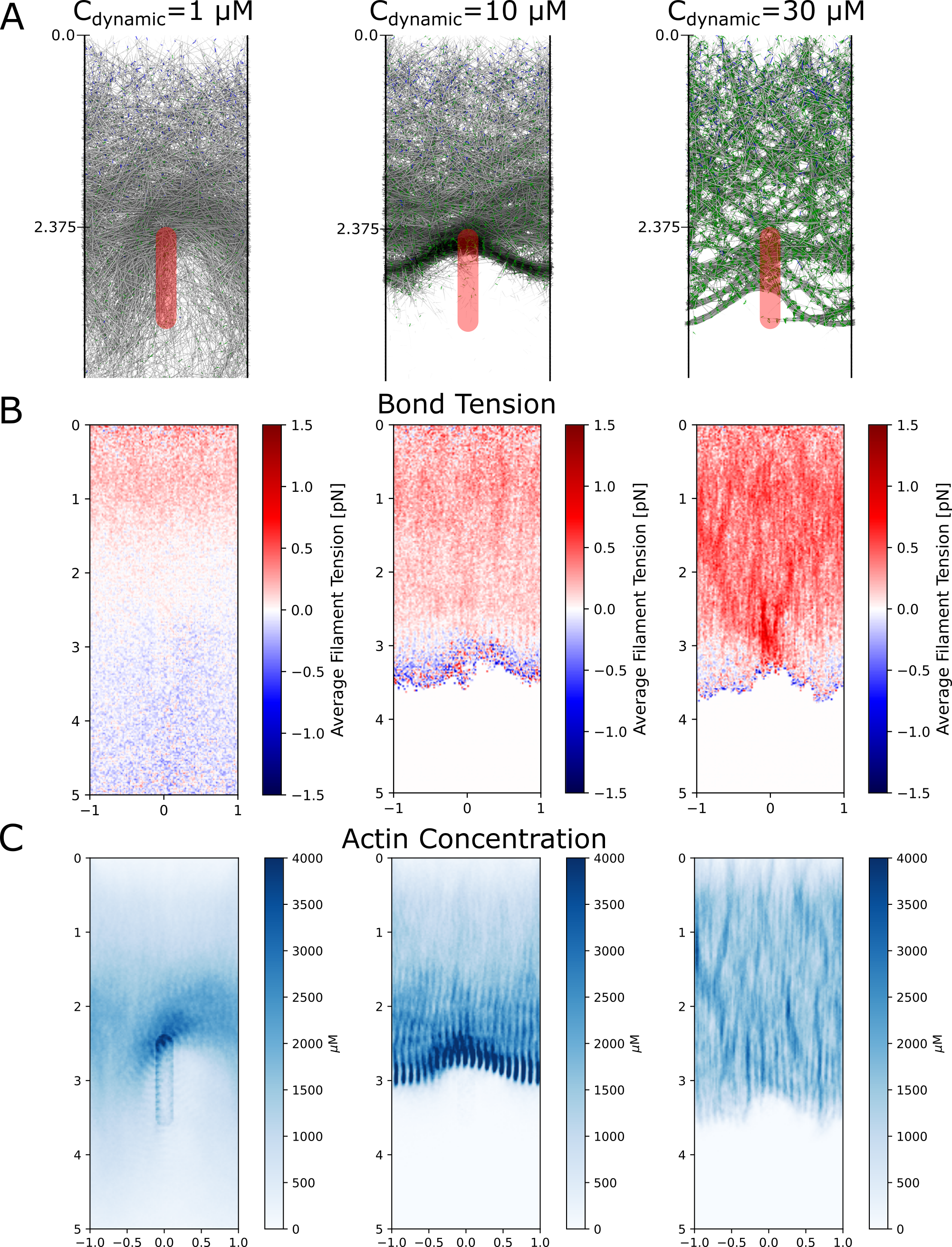

Supplement: S2 Fig — (A) Images of actin network at different dynamic crosslinker concentration, same as in Fig 6B, with a combination of long-lived and dynamic crosslinkers and focal adhesion representing a mature adhesion. Uniform pulling was applied. Blue line segments represent permanent crosslinkers while green line segments represent dynamic crosslinkers. (B) Tension between actin filament beads (averaged over both time and the z-axis at steady state) for the corresponding simulations in A show that the actin filaments are mainly under compression. (C) Actin concentration values (averaged over both time and the z-axis at steady state) for corresponding simulations in A. The scale along the vertical (y) and horizontal (x) axes is in μm. (TIF) [file pcbi.1009506.s002.tif]

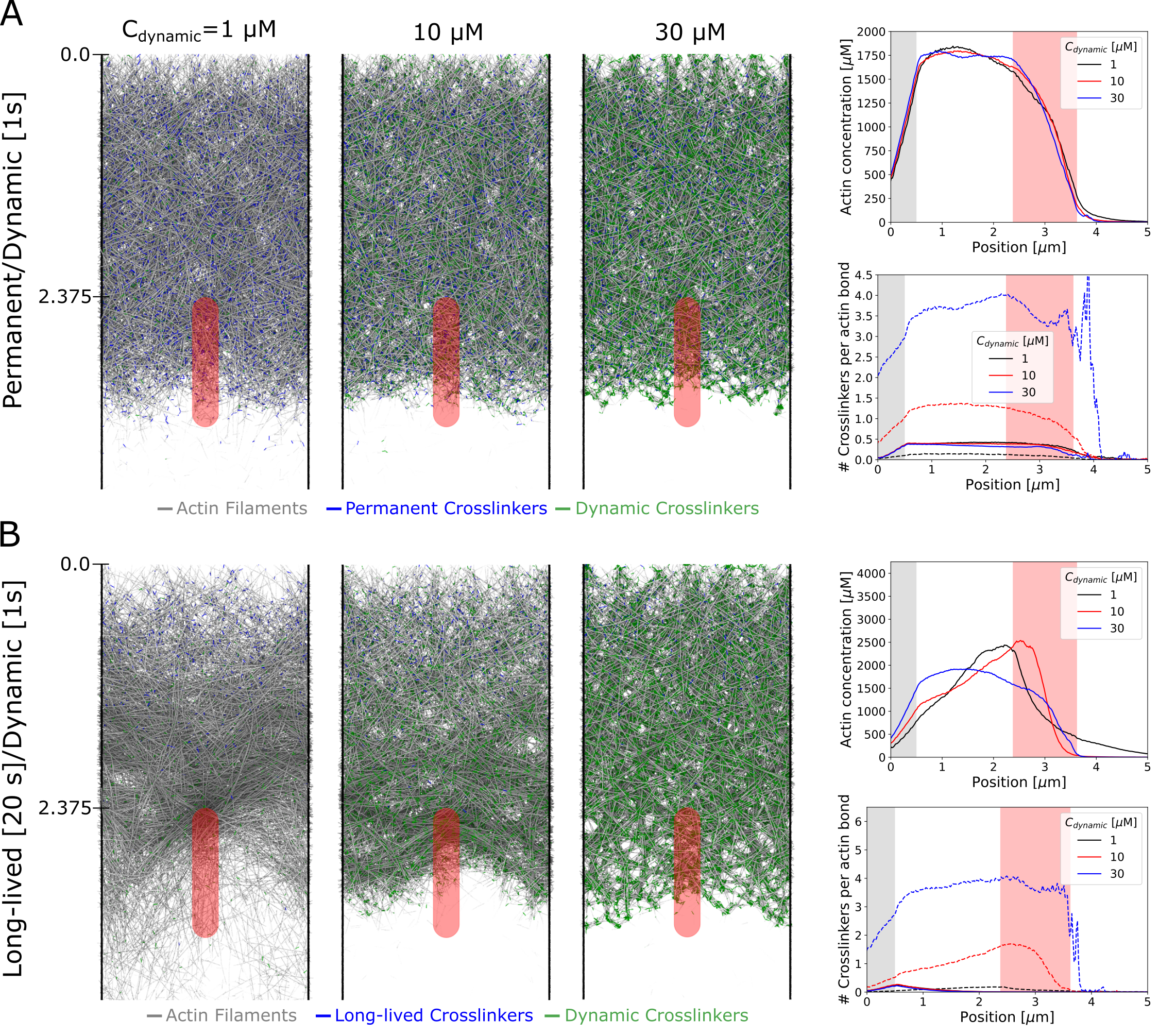

Supplement: S3 Fig — (A) Snapshots (left) and concentration profiles (right) by a combination of permanent and dynamic crosslinkers. Increasing the number of dynamic crosslinkers results in tighter bundles of filaments while leaving the overall spatial concentration of filaments relatively unchanged and there is no bundling of filaments near the focal adhesion region. Blue (green) line segments represent permanent (dynamic) crosslinkers. (B) Same as panel A but replacing permanent crosslinkers with crosslinkers that have a finite lifetime of 20 s. Bundling, together with a peak in actin concentration, occurs in front of the focal adhesion region at intermediate dynamic crosslinker concentrations. The resulting bundle is less compact compared to the uniform pull case (Fig 6B). In the simulation snapshots, the scale along the vertical (y) axis is in μm. Blue (green) line segments represent long-lived (dynamic) crosslinkers. All data calculated from simulations equilibrated for at least 165 s and averaged over at least 24 s. (TIF) [file pcbi.1009506.s003.tif]

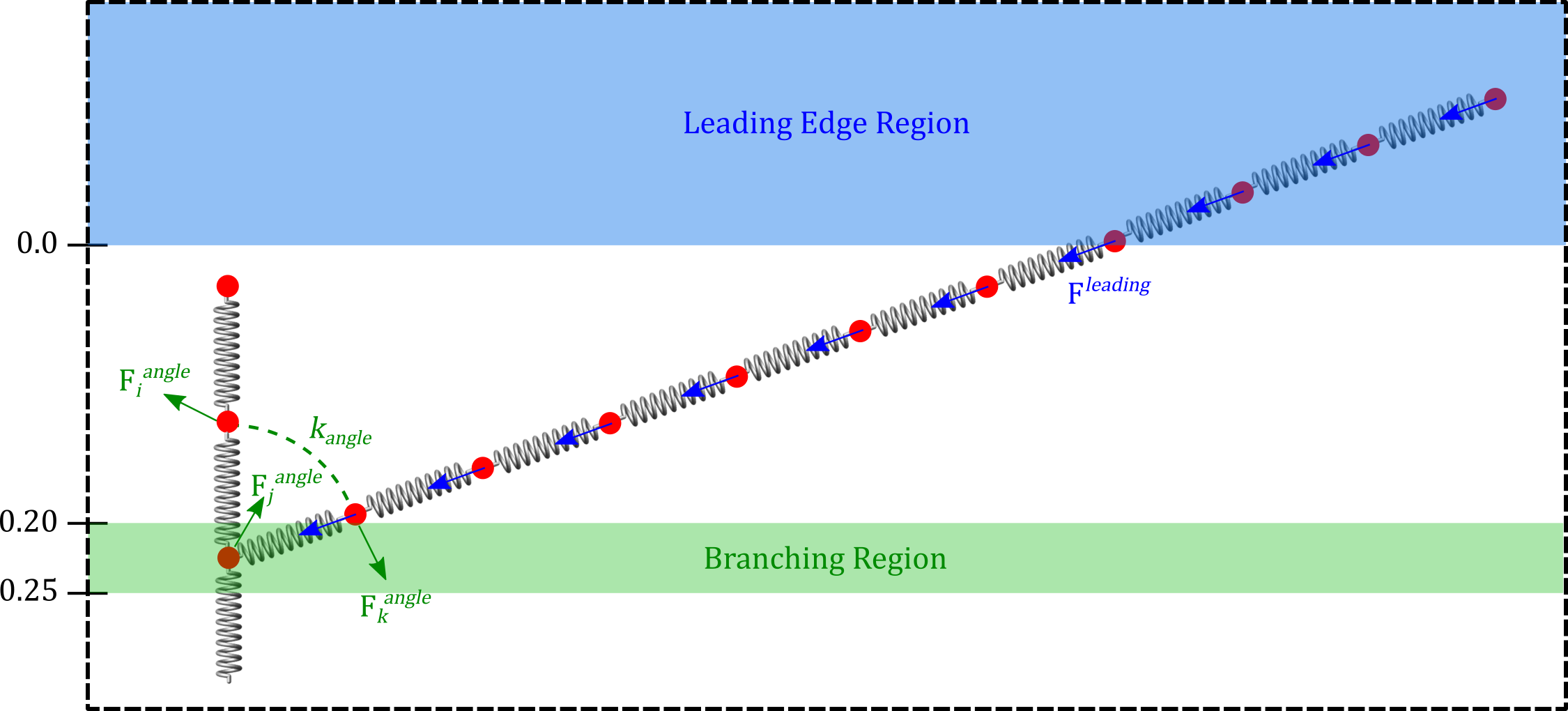

Supplement: S4 Fig — In the branching simulations of Fig 8, branches are added to a randomly selected actin bead between y = 0.25 and y = 0.20 μm, highlighted by the green region. The branch is held at a prescribed angle of 70° by an angular force, Fangle, defined in Eq 6, which acts on the three beads comprising the angle. Pushing forces from the leading edge shown in blue act on all beads of the daughter filament. In this example, there are no pushing forces on the mother filament beads to which the daughter filament is connected because the mother filament does not have a bead extending into the leading edge region. (TIF) [file pcbi.1009506.s004.tif]
