# Supplementary material for: Discrete mechanical model of lamellipodial actin network implements molecular clutch mechanism and generates arcs and microspikes
Source: PLoS Comput Biol. 2021 Oct 18;17(10):e1009506. doi: 10.1371/journal.pcbi.1009506 (PMC8553091; doi:10.1371/journal.pcbi.1009506)
Supplement: Attachment — Submitted filename: ResponseToReviewers.pdf [file pcbi.1009506.s010.pdf]

Response to Reviewer 1.

Reviewer #1: This manuscript introduces a well-defined model for simulating a stationary lamellipodium. The model is primarily based on Brownian dynamics and includes explicit representation of individual actin filaments and a nascent focal adhesion. This allows them to investigate local deformation and force development with a retrograde flow at steady state. Most of the results are consistent with previous experimental findings. However, there are still some parts that lack explanations. The comments are listed below.

A: Thank you for the positive assessment of our work and for the feedback.

By providing a filament-level representation of the lamellipodium, which goes beyond prior continuum models, our work opens the way to ask mechanistic questions at an even finer level of detail. Indeed, this was our goal and the scope of many of the following comments by the reviewer. As we explain below, we feel we must leave the answer to some of these questions for future work, either because we lack mechanistic information or because we felt these questions can be better answered by first completing a model at the current level of detail. We did however consider modifications/refinements to our model to address the comments and performed additional simulations as described below.

< Major comments >- It is assumed that permanent cross-linkers can represent Arp2/3. However, there is a structural difference between a branched formed by Arp2/3 and a cross-linking point between two filaments. In particular, it is expected that 70 deg formed by Arp2/3 is maintained by intrinsic bending stiffness. Torsional rotation would not be allowed. However, it seems that the cross-linkers in the model do not have such things. This may affect the mechanical and dynamic behaviors of the lamellipodium significantly. The authors should justify the use of a simple cross-linker for Arp2/3.

A: As mentioned by the reviewer, there is no angular restriction to represent branching at 70 degrees in our simulations.

We note that the lamellipodial network undergoes a structural change from a branched network close to the leading edge to a network of longer filaments further back (through unknown mechanisms; we have recently proposed a model that includes filament severing and annealing in Holz et al. bioRxiv https://doi.org/10.1101/2021.03.31.437985). We had postponed addressing the spatially-varying branching architecture in this paper, opting for an approximate, intermediate, description that represents the average behavior between the branched and linear regions.

To address the accuracy of this approximation, we added branching to our model and performed additional simulations with uniform pulling shown in new Figure 8, S4, and new section “Robustness of model to variations”. We perform simulations where new daughter filaments of 1 μm in length are introduced as permanent branches off mother filaments close to the leading edge as shown in new Figure S4. The angle between the

mother and daughter filament is restricted to near 70 degrees by an angular force. We find that the qualitative as well as quantitative features of these simulations are not that different from the simulations with permanent crosslinkers: a network made of branches behaves similarly to a network with permanent crosslinkers in terms of retrograde flow profiles, density profiles, and force profiles (Figure 8), with small differences as expected from networks with similar concentrations but somewhat different connectivity. This means that our simulation is a useful starting point to further explore the branching biomechanics.

We did not yet add a restriction on the torsional rotation of filaments, which we believe should be addressed in future work. The effect of filament torsion in our model should be evident at the level of individual branches rotating around their mother filament. This rotation however, is likely strongly suppressed already due to the thin dimension of our simulation box perpendicular to the branching plane and the additional aspect of steric hindrance due to neighboring filaments. This is suggested from snapshots of these branching simulations where the vast majority of branches are seen along the xy plane (Figure 8C). To explain the lack of torsional restriction even in these new simulations with branches we have added the following sentences to the newly added branching section of the Modeling methods section: "Torsional rotation of daughter filaments around their mother filament is not constrained by this angular potential, but torsional rotation is restricted by the thin dimensions of our simulation box perpendicular to the branching plane as well as excluded volume interactions among filaments. This restriction is suggested from snapshots of branching simulations in Fig 8C where the majority of branches are in the xy plane even far from their introduction near the leading edge."

- Cross-linkers can bind only to actin beads, which means that distances between cross-linkers would be the multiple of 100 nm. This also means that the length of "branches" in this pseudo-branched network structure would be also the multiple of 100 nm. However, in real branched structures formed by Arp2/3, this is far from true. The authors should provide justification about the assumption of the binding between actin filaments and cross-linkers.

A: The reviewer is correct that cross-linkers and branches can be spaced closer than 100 nm. In particular, actin filaments have a helical repeat every 36 nm. Since branching occurs predominately along the xy plane in the lamellipodium, Arp 2/3 branches could form as close as 36 nm along filaments [as observed by Vinzenz et al. J Cell Sci 125:2772 (2012)]. To investigate this issue in more detail we ran simulations where the actin beads are spaced closer together than our original simulations, at $l_0 =$ 48.6 nm (18 actin monomers), approximately half of the number of monomers between actin beads in our original simulations as discussed in the new "Robustness of model to variations" section. As with the branching simulations described in the previous comment, we ran these simulations with uniform pulling at $\kappa_{FA} = 100$. To compensate for this higher number of filament beads, we reduced the drag coefficients and pulling and pushing force per bead by half. To compare to our reference simulations with $l_0 = 100\ nm$, we measured the retrograde flow speed, density, and compression/extension

of the network (Fig 8). Again we find that the qualitative as well as quantitative features of these simulations are not that different from the simulations with permanent crosslinkers. The following small differences can be explained by an increase in the number of permanent crosslinkers (by ~20% over the $l_0 = 100$ nm simulations) as a result of the higher cross-linking opportunities during the filament insertion process:

- The retrograde flow speed is slightly lower than in the $l_0 = 100\ nm$ simulations (Fig 8A), leading to a slightly higher actin density in front of the focal adhesion, but a lower density behind the focal adhesion (Fig 8B).
- There are higher levels of filament extension behind the focal adhesion in the $l_0 = 48.6\ nm$ simulations (Fig 8D).

Future work could include a more accurate representation of the filament helix, which should be preferable to allowing cross-linkers to be placed anywhere and at all angles along the filament (e.g. Bidone et al. PLOS Comp Biol 12:e1005277 (2017) allowed for a spacing of 7 nm while the method of Nedelec and Foethke New J Phys 9:427 (2007) allowed for arbitrary placement of crosslinkers along filaments).

- The focal adhesion is assumed to be a non-dynamic structure without further maturation or a decrease in size. Then, it would just behave as a friction pad to the moving network rather than a thing that behaves as a mechanosensitive structure as well as a friction pad. This might have been a convenient, simple setup for the model, but it doesn't account for the reality well. How would the dynamic focal adhesion change the results overall?

A: The reviewer is correct that the focal adhesion in our model is not a dynamic structure and only acts as a region of increased friction. In future simulations we plan to incorporate a more complicated focal adhesion model, possibly involving discrete talin molecules that unfold upon the application of force. However, since we focus on determining the steady state behavior of the actin network in this paper, we did not want to complicate the model with inclusion of a dynamically changing focal adhesion region. A dynamic focal adhesion would likely lead to longer simulation equilibration times to steady state, and should lead to reduced retrograde flow speed with higher compression in front of the focal adhesion region as the focal adhesion matures. We have added the following sentences to the Discussion section: "The model for the focal adhesion could also be improved by allowing it to change in size/shape in response to forces exerted on it by the actin network. One way of implementing a more complex focal adhesion is to incorporate discrete talin and vinculin molecules recruited to the focal adhesion region as a function of force."

- In the last paragraph of the introduction (page 3), the authors should briefly provide some numbers or descriptions of the model and elaborate the quantitative definition of "wide, steady lamellipodia" as well as the speed of retrograde flow. Are these numbers dependent on cell types? What is the standard or criterion for their stability in terms of size, morphology, and lifetime?

A: Thank you for this suggestion, we have added some numbers and better and expanded upon the selection of XTC cells as a reference case (see last paragraph of Introduction).

- What is the boundary condition in z-direction? Further, the orientations of filaments in the xy plane are not normally distributed. As the authors mentioned, this simplifies the force generation by polymerization or membrane tether. However, as far as I understand, the model does not account for a plasma membrane component. How would that affect the average pushing force exerted on each filament (~15 pN) measured from experiments? Also, what is the reference for the 15 pN?

A: A reflecting boundary condition is imposed in in the z-direction by applying a constant force of 1 pN in the z-direction, to any bead crossing the boundary, towards the interior of the box. This is mentioned at the end of section "Crosslinker, excluded volume, and confining forces" of the Modeling methods section. This force is meant to represent the effect of the plasma membrane by restricting the actin beads to be within a simulation box of height 0.2 μm (approximate height for the lamellipodium).

We are sorry, but the value of the pushing force per filament was misreported as 15 pN when it should have reported as 1.5 pN. This has been corrected where present throughout the manuscript (all other force results reported, including Fig. 4, were correctly reported and remain unchanged) This force per filament is on the order of theoretical and experimental estimates of force produced by actin polymerization (Mogilner and Oster Biophys J 71:3030 (1996); Kovar and Pollard PNAS 101:14725 (2004)) and similar to the estimated stall force in keratocytes of 1.7 pNs per filament (Heinemann, Doschke, Radmacher Biophys. J. 100:1420 (2011). We updated the discussion in the "Membrane and motor pulling forces" section accordingly.

We have chosen a simplified description of the boundary condition at the leading edge, where the effect of the plasma membrane on the network is only included in the generation of a pushing force for filaments at the leading edge. The force-generating properties of membrane-attached complexes that transfer actin or profilin-actin to the barbed ends of filaments would have a complex dependence of force on filament length and orientation (e.g. as in the elastic Brownian ratchet model of Mogilner and Oster). The current and rather limited scope of our simulations is to capture the net effect of these processes. More realistic implementations of the plasma membrane may allow for more interesting / complex networks than the ones we find here, including for the possibility of filopodia (filament bundles protruding beyond the leading edge) embedded in the lamellipodia. We updated the third paragraph of the Results to clarify some of the above points.

- In Figure 1, it would be better to illustrate the motor force and crosslink forces. Schematic representation of motors is missing too. It might be difficult to draw the leading force in the force balance equation, but a simple demonstration would still be helpful.

A: Thank you for this suggestion. We have added cartoon images of the motor forces, crosslinking forces, and the leading edge force to an updated version of Figure 1 that is now in the manuscript.

- What are the simple criteria to differentiate the motors that exert uniform force from those that generate back force? Are all beads farther than 3.25 μm from the leading edge subjected to back pulling forces? What is the relative magnitude of the two forces?

A: We are sorry for confusion on the motor pulling, but hopefully the additions to Figure 1 should help with the explanation. The answers to these questions are found in the document in the "Membrane and motor pulling forces" sub-section of the Modeling methods section. The motors, regardless of whether they are pull uniform or pull back, are implemented in a continuum manner and only act on beads that are in a certain spatial region. For back pulling forces, every bead further than 3.25 μm from the leading edge is subjected to back pulling forces of 0.004 pN/filament bead. The motors in the pull uniform case are half the magnitude as in the pull back case (0.002 pN/filament bead).

The filament segments are removed by severing near the bottom of the network as they mature. The simulation is run for ~65 s as mentioned in the method section, but the severing is for aged filaments with a lifetime longer than 125 s. Is there a discrepancy?

A: We are sorry for the confusion here. The value of 65 s was meant to indicate that by running the simulation on XSEDE's Comet for 48 hours, we generated approximately 65 s of simulation time. The majority of simulations were run for simulation times several times this reported 65 s (at least two times the average filament segment lifetime of 125 s). To clarify this issue, we have added the simulation time at which a given image/data was taken to the figure captions of Figures 2, 3, 6, 7, 8, and S3. We also added the following sentence to the Simulation subsection of the Modeling methods section "To reach steady state, simulations were typically restarted around five times meaning that the total simulation time was typically around 300 s."

- In Figure 2A, for both pull back and pull uniform cases, there is always a sharp increase in magnitude as well as fluctuations for the retrograde velocity far from the leading edge, what is the explanation for that? Is it due to a variation in density of actin filaments? It would make a sense in the case of highest focal adhesion strength where there is a reduction in density as shown in 2D, but the reference case does not have such a reduction in density.

A: The reason for the increase in magnitude and fluctuations of the retrograde flow velocity is that in this region the lamellipodium fragments into disconnected pieces.

These pieces have varying drag and diffusion coefficients that get pulled away from the connected region. In cells, the region behind the lamellipodium would be occupied by the lamella and this increase in retrograde flow would likely not occur, but our model cannot currently represent this region accurately. We have clarified this in the third paragraph of "Actin structure and dynamics affected by presence of focal adhesion".

- It is interesting that the authors did not find significant perturbation of local network density or velocity by the nascent focal adhesion. However, in vivo, the strong correlation between motility force and adhesion/density is very commonly observed during cell migration. (i.e., https://www.pnas.org/content/118/4/e2009959118). This might be due to the lack of stress fiber structure as well as maturation of focal adhesion. Further discussion might be helpful.

A: We thank the reviewer for providing this reference (Schreiber et al.) which helped us to better place our results in the larger context of cell motion.

We do find that increase of the focal adhesion strength (parameter κ_{FA}) leads to a global reduction of retrograde flow, which is consistent with the basic claim of the clutch model (Fig 2A). The pushing force is also higher at larger adhesion strengths (Fig. 4), which could result in motion if membrane flow allows for protrusion. By "local" perturbation we mean the region around the nascent focal adhesion itself (a region of size ~ 0.25 μm). That we don't see significant local perturbations is consistent with the experimental observation that nascent focal adhesions do not locally perturb the velocity field around the region of the focal adhesion in XTC cells [Yamashiro et al. MBoC 29:1941 (2014)]. To address this comment, we changed the 3rd and 4th paragraphs of section "Actin structure and dynamics affected by presence of focal adhesion" accordingly.

Our model cannot describe how global cell motion is affected by adhesion density or adhesion maturation since we do not include traction by lamella, stress fibers, or myosin II contraction. The paper by Schreiber et al. discusses the universal observation of biphasic dependency of cell velocity on ligand density. We modified the fourth paragraph of the Dicsussion to clarify this, which includes the reference suggested by the reviewer.

- Is it possible to add more points to make the curves in Figure 4 more continuous? This is also applicable to Figure 5A. Plus, would it be necessary to perform some averaging of the data in each condition (with multiple runs)?

A: The averages for the data in Figure 4 and 5A are taken after the system has equilibrated and reached steady state. We limited the number of data points because these simulations are computationally expensive. They were however averaged over a significant amount of time at steady state, where the relative error bars should be small. To check the level of accuracy of these measurements, we reran simulations in the pull uniform case for $\kappa_{FA} = 100$ and $\kappa_{FA} = 500$ and included the additional data points in

Figure 4. From these simulations we do not find a large change in the values in Figure 4 in between simulations, as we expected. We did not perform additional simulations as we do not see a reason for certain values of κ_{FA} to show higher variance while the curves in Figure 5A should be monotonic and are largely linear in the range of κ_{FA} values.

- If the two modes of pulling force lead to such a minimal difference in terms of the development of viscous and external forces as well as retrograde flow speed, then is it better to reconsider the initial assumption of the two pulling modes? Also, the switch of tension to compression is the same in both cases. The effect is negligible. Are the results in Figure 6 independent of the pulling modes as only the uniform pulling is implemented?

A: As the nature of the motors guiding retrograde flow is not firmly established, we thought it is important to show both uniform and back pulling modes, even if both end up giving similar results (a result that is not otherwise obvious). The results in Figure 6 were only originally run in the uniform pulling mode, but in response to this comment, we also ran these six simulations in the pull back mode and display the results in supplemental figure Fig S3. The results in Fig S3 show that as networks becomes less crosslinked, there is a more noticeable difference between pull uniform and pull back modes: In Fig S3B where the permanent crosslinkers are replaced by crosslinkers with a finite lifetime of 20 s, we see that for simulations at $C_{dynamic} = 10$ μM the arc formed near the focal adhesion region is less sharp, being more spread out by the assumed higher back pulling forces in that region, We discuss the implications of this observation in the last paragraph of section “Arc formation at the lamellipodium boundary”.

- The authors showed the formation of actin arcs near the focal adhesion. However, the actin arcs have been observed at the interface between lamellipodia and lamella without large focal adhesions. It seems that dense myosin population at the interface induces such arc formation rather than focal adhesion. Nevertheless, the observation of arc formation in this study was highlighted even in the title. The authors should demonstrate that a relationship between arc formation and focal adhesion by citing enough experimental studies.

A: The mechanism of arc formation in Fig 6 is similar to the mechanism proposed theoretically by Shemesh et al. Biophys. J. 97:1254-64 (2009) where filaments align and compact near focal adhesions. Transverse actin arcs can form at the lamellipodium-lamella interface, which is where focal adhesions also mature. However, upon closer examination of the literature as suggested by the reviewer, we have not been able to find a direct experimental link between focal adhesions and arc formation (quite possibly, not something that has not been investigated in great detail yet).

We thus rephrased our discussion of Fig. 6 as indicated by red color in the section with new title “Arc formation at the lamellipodium boundary”. We kept the reference to actin

arcs in the title of the paper since a reduction of retrograde flow at the lamellipodium/lamella interface, whether mediated by large focal adhesions or not, would lead to arc bundle formation by the same mechanism. We further now state that this mechanism of filament alignment and compaction may act in conjunction with (or help trigger) myosin-II to form transverse arcs and the two mechanisms do not need to be mutually exclusive. Though we do not simulate discrete myosins, the dynamic crosslinkers in our simulations may be playing a similar bundling role. We also added references to the related phenomenon of arc formation through microspike aggregation occurring even when myosin-II is inhibited by blebbistatin [Koestler et al. Nat. Cell Biol. 10:306 (2008), Nemethova et al. J. Cell Biol. 180:1233 (2008)]. Indeed, we have been able to reproduce this phenomenon in recent simulations that we decided to leave for future work. Please also see our response to the final comment of Reviewer 2.

< Minor comments >

- Page 3, Line 59: "many have simplifying assumptions and do not include discrete filament force…" -> "simplifying" should be "simplified". This should also be corrected in the rest of the manuscript.

A: We have changed all instances of "simplifying" to "simplified"

- Page 3, Line 77: "Here we model of a piece of the lamellipodium actin network at the filament level" -> "model of" seems to be wrong grammatically.

A: Thank you for noticing this issue. We rewrote the beginning of this sentence as "Here we model a 2 μm wide strip of the lamellipodium…"

- Page 3, Line 92: "filopodia" -> spelling. (Also, Page 15, Line 376).

A: We have fixed the spelling in both locations.

- Page 13, Line 336: "with their axis along", possibly better to use "with their axes along".

A: We have fixed the spelling here.

Response to Reviewer 2.

Reviewer #2: This is a very interesting study by Rutkowski and Vavylonis, where they used coarse-grained molecular dynamics simulations to investigate the emergence of different actin network organizations within a steady-state lamellipodium. The authors used the well-studied XTC cells to benchmark their simulations in order to study the relationship between retrograde flow speed, focal adhesion strength, and force generation in lamellipodial actin networks. One of the most important findings are the conditions under which microspikes, actin arcs and bundles emerge in actin networks. Especially the emergence of lamellar arcs and stress fibre like architectures are novel. Although the results are well presented with high quality figures and movies, there are shortcomings in the clarity of explanation and comparison with experiments. I therefore suggest the authors address the following points in a suitably revised manuscript.

A: Thank you for the positive assessment of our work and for the feedback. We tried to address these points as described below.

- It is unclear how the authors deduce the molecular composition of the actin network from experimental observation. For instance how do the authors choose the density of actin, relative abundance of Arp2/3 and dynamic crosslinks? How are these choices grounded in experimental observation.

A: In the last paragraph of the "Actin structure and dynamics affected by presence of focal adhesion" section of the Results section we had mentioned that the actin density is "in the range of 800 μM (dashed line in Fig 2C), which is the typical value for the density of the lamellipodial actin network [Watanabe Proc. Jpn. Acad. Ser. B Phys. Biol. Sci. 86:62 (2010)]".

The concentration of Arp2/3 complex in XTC cell lamellipodia was estimated to be 2.3 μM [Tsuji et al. Plos One 4:e4921 (2009)], which corresponds to one Arp2/3 complex per 0.9 μm of an actin filament, close to the value of 0.75-0.8 per μm observed by electron microscopy of fibroblast lamellipodia [Vinzenz et al. J Cell Sci 125:2772 (2012)]. Average actin filament length in the branch network within a μm to the leading edge however can be as small as 0.16-0.2 μm [Vinzenz et al. 2012; Bailly et al. J. Cell Biol. 145:331 (1999)]. In a typical simulation with κ_{FA}=100, we created permanent cross-links with an average density of one per 0.3 μm, a value in between these two limits. With this value we obtain networks that have an elastic modulus comparable to experimental values, even though we may have a somewhat larger density of permanent cross-links than what would correspond to just the Arp2/3 complex. We note that in response to a question by Reviewer 1, we ran new simulations with branches instead of permanent crosslinkers, but we do not find much difference in terms of retrograde flow profiles, density profiles, and force profiles (as seen in the new Figure 8).

To clarify this point we added the following to the 3rd paragraph of the “Network generation, crosslinking, and disassembly” section of the Modeling methods section:

“The concentration of Arp2/3 complex in XTC cell lamellipodia was estimated to be 2.3 μM [Tsuji et al. Plos One 4:e4921 (2009)], which corresponds to approximately one Arp2/3 complex per 0.9 μm of an actin filament, close to a branch distance of 0.75-0.8 per μm observed by electron microscopy of fibroblast lamellipodia [Vinzenz et al. J Cell Sci 125:2772 (2012)]. Average actin filament length in the branch network within a μm to the leading edge can be as small as 0.2 μm [Vinzenz et al. 2012; Bailly et al. J. Cell Biol. 145:331 (1999)]. In a typical simulation with κ_{FA}=100, permanent cross-links have an average density of one per 0.3 μm, a value comparable to these experimental measurements.”

The dynamic crosslinker concentration in our simulations includes the effect of α-actinin, filamin and possibly plastin. Both filamin and α-actinin have individually a lower concentration in the lamellipodium than Arp2/3 complex as determined from fluorescence measurements fibroblast lamellipodia [Svitkina and Borisy J. Cell Biol. 145:1009-26 (1997)]. In agreement with this estimate, the molar ratio of filamin:actin is 1:115 and α-actinin:actin at 1:50 in macrophage cells [Pollard and Cooper Annu. Rev. Biochem. 55:987-1035 (1986)]. Using an actin concentration of 100 μM for the entire cell, the sum of the α-actinin and filamin concentrations should then be 2.9 μM, which is within the range that we investigate in this paper of 1-30 μM (as defined within the whole simulation box). To clarify this point we added the following to the 3rd paragraph of the “Network generation, crosslinking, and disassembly” section of the Modeling methods section:

“In our simulations, we vary $C_{dyanmic}$ between 1 and 30 μM, a range containing an estimate of the net α-actinin and filamin crosslinker concentration of 2.9 μM, determined by using 100 μM actin concentration for the entire cell and the experimentally determined α-actinin and filamin crosslinker to actin molar ratios in macrophages [Pollard and Cooper 1986].”

- Typically myosin-II minifilaments are excluded from lamellipodial region and are found in the lamellar region. Therefore the case of uniform pulling does not make biological sense. Could the authors justify this choice? I am not aware of any experimental data that show contractile motors in the lamellipodium. Its also unclear why the motor pulling forces are acting vertically downward. Shouldn’t they act parallel to the filaments which will lead to both vertical and horizontal components?

A: Motors that contribute to the retrograde flow (that remains even after inhibition by blebbistatin in XTC cells [Yamashiro et al. Mol. Biol. Cell 25:1010 (2014)]) could be motors which associate with the plasma membrane like certain myosin I motors have been known to do [Woolner Trends Cell Biol. 9:245 (2009), McIntosh J. Cell Sci. 129:2689 (2016)]. The pull uniform mode was an attempt to include the effect of these myosin I motors, in a general way, as mentioned in the 6th paragraph of the Discussion section. Motivated by the reviewer’s comment, we investigated this assumption in more

detail in a new Fig. 8 where we only pull actin beads that are within 20 nm of the bottom of the simulation box (representing the membrane adhered to the external substrate), outside the focal adhesion region. As mentioned by the reviewer, representing motor force as a vertical force is likely not accurate and so in these membrane pulling simulations the pulling force is directed along the axis of the filaments instead of along the direction of retrograde flow. Regardless of these changes, using a pulling force of 10x that of the original pulling force (0.02 pN per actin bead), these simulations result in comparable values of the retrograde flow, network density and morphology, and average filament tension to the pull uniform simulations at the same focal adhesion strength (Fig 8A,B,C,D).

- Retrograde flow speed depend both on motors forces and actin polymerisation. How are their relative contributions modelled?

A: We tuned the magnitude of the motor force such that the retrograde flow was slightly more influenced by polymerization forces than motor forces under a wide range of focal adhesion strengths. This is suggested by experiments of cells treated by cytochalasin D [Henson et al. Mol. Biol. Cell 10:4075 (1999), Alexandrova et al. PLoS One 3:e3234 (2008)] mentioned in the beginning of section “Response to inhibition of polymerization”, showing a greater than 50% reduction in retrograde flow without complete abolishment.

To further clarify the balance between polymerization and motor forces we have added the following to the fifth paragraph of the Results section “We aimed for a slightly higher contribution of the leading edge force than the motor pulling force to retrograde flow, as suggested by experiments halting acting polymerization with cytochalasin D [Henson et al., Alexandrova et al.] and discussed in section``Response to inhibition of polymerization'' below.”

We also modified/added the following to the “Response to inhibition of polymerization” section:

“In these simulations on the effects of cytochalasin D, the retrograde flow speed decreases to approximately 55% of the initial value when under back pulling and to 45% under uniform pulling at κ_{FA}= 1 (Fig 5A). As the focal adhesion strength increases, the retrograde flow decreases by more than 50% after ceasing filament addition, suggesting that the pushing force in these cases was larger compared to the pulling force at steady state (as shown in Fig 4B). This dependence of the degree of reduction of retrograde flow on adhesion strength could explain why the retrograde flow was reduced to a different level after cytochalasin D treatment in prior experimental studies [Henson et al., Alexandrova et al.].”

- In Fig. 2 it is not surprising that in the focal adhesion region actin flow is reduced. The result is somewhat obvious since viscosity is increased at the sites of adhesion.

A: The reviewer is correct, the slowdown of retrograde flow with increasing focal adhesion strength is not particularly surprising as it only indicates that there is increased resistance to flow on the actin network. What may be less obvious is that the local density and retrograde flow (around the adhesion) are relatively unperturbed.

- The authors find that the F-actin flow profile (Fig. 2) is non-monotonic. Could they compare this to existing experimental data? Various groups have measure F-actin flow profiles in lamellipodia.

A: As mentioned in the third paragraph of the “Actin structure and dynamics affected by presence of focal adhesion” section of the Results section, the retrograde flow profile is largely constant in the lamellipodium of XTC cells [Yamashiro et al. MBoC 29:1941 (2014)]], which is consistent with our results. The region near the beginning of the lamellipodium (less than y = 1 μm) reflects the boundary condition of filament insertion and is not meant to be compared to experiments. The region at the end of the lamellipodium shows an increase in magnitude and fluctuations of retrograde flow because in this region the lamellipodium fragments into disconnected pieces. These pieces have varying drag and diffusion coefficients that get pulled away from the connected region. In cells, the region behind the lamellipodium would be occupied by the lamella and this increase in retrograde flow would likely not occur.

We have clarified these issues in the 3rd paragraph of section “Actin structure and dynamics affected by presence of focal adhesion”

We note that in certain cell types, for example Ptk1 cells in Craig et al. Phys Biol 12 (2015) 035002, the retrograde flow decreases by a large fraction through the lamellipodium, as function of distance to the leading edge. However, these cells have much narrower lamellipodia (and thus have quicker transition to the lamella region) as compared to the lamellipodia of XTC cells that we use as a reference. In addition, the retrograde flow of XTC cells has been measured very accurately by single molecules.

- The authors say "While the network velocity and density are not significantly perturbed locally by the 218 presence of a nascent focal adhesion” - Fig 2 seems to suggest otherwise

A: We are sorry for the confusion. We have updated the discussion of Figure 2 in the third and fourth paragraphs of section “Actin structure and dynamics affected by presence of focal adhesion” where we now better distinguish (i) the global reduction of retrograde flow of Fig. 2A by the focal adhesion as a function of focal adhesion strength and (ii) the much smaller 2D local spatial variations around the focal adhesion for a given adhesion strength. We also added a reference to the panels of Fig 2 (2B and D) in the sentence mentioned by the reviewer (first sentence of section “Force distribution affected by focal adhesion”).

- How are pushing and pulling forces computed in Fig 4? I thought these were input parameters to the model (as defined in Fig. 1)

A: We are sorry for the confusion here. The pushing and pulling force per bead are indeed input parameters, but since the fraction of actin beads in the pushing or pull back region changes as a function of focal adhesion strength, the forces exerted by the pushing or pull back force on the entire actin network also changes as seen in Fig 4. The pull uniform force is constant in Fig 4 since it acts on every actin bead in the system and the number of beads is fixed by the insertion and turnover rates.

We had tried to explain this in the third paragraph of the “Force distribution affected by focal adhesion” section:

“As the focal adhesion strength increases, the pushing force increases because there are more filaments in the pushing region and filaments are inserted at a fixed constant rate.… In contrast, in the pull back case, the pulling force decreases with increasing focal adhesion strength since there are fewer actin beads in the pull back region. ”

We now added a better explanation of how the forces were calculated in the legend of Fig. 4:

“Forces in these plots are the net force on the actin network divided by either the leading edge width of 2 μm (force per μm) or per number of actin beads in the simulation (force per bead)”.

- I found the results in Fig. 6 and 7 very interesting, especially how actin bundles emerge within a disordered network. The authors may want to discuss these results in light of recent experimental studies - Vignaud et al Nature Materials 20:410 (2021), Lehtimaki et al eLife 10:e60710(2021). How do the length of the bundles in Fig. 7 compare with the typical size of microspikes?

A: We thank the reviewer for pointing out the relationship of our simulations to these recent works. We added the following paragraph at the end of section “Microspike formation within lamellipodia”:

“The formation of bundles within our simulated networks in both Fig 6 and Fig 7 is reminiscent of actomyosin bundle formation within a disordered cortical actin network [Vignaud et al. 2021; Lehtimaeki et al. 2021]. While we do not explicitly model myosin motors, our simulations show how dynamic or permanent passive crosslinkers, in combination with actin turnover and alignment through flow (Fig 6) or polymerization (Fig 7) can promote and maintain bundled structures within disordered networks.”

The length of the simulated bundles in Fig 7 is comparable to the lamellipodium size, similar to experimental images of microspikes that have lengths ~4-5 μm. Microscpikes

in cells are often oriented at an angle to the leading edge [Koestler et al. Nat. Cell Biol. 10:306 (2008), Nemethova et al. J. Cell Biol. 180:1233 (2008)]. When these microspike structures encounter the slower moving lamellum they occasionally collapse and are incorporated into arcs with orientation parallel to the leading edge [Nemethova et al. J. Cell Biol. 180:1233 (2008)]. We have been able to reproduce such tilted and collapsing microspike dynamics by a few additional changes to our simulations (for example by insertion of filaments with a bias at ±35 degrees), which we plan to report in future work.
